# Letters to the Editor

**DOI:** 10.1101/gad.350482.123

**Published:** 2023-01-01

**Authors:** James T. Kadonaga

**Affiliations:** Department of Molecular Biology, University of California, San Diego, La Jolla, California 92093, USA

Dear Terri, it is a very special privilege and honor for me to write this note of thanks for your lasting and impactful contribution to the advancement of science through your 35 yr of service as the Editor of *Genes & Development* (which we all refer to, of course, as “*G&D*”). Under your leadership, *G&D* quickly and very impressively rose to the top tier of journals in the biological sciences, and it has published countless landmark papers. I am very fortunate to be a coauthor on 28 *G&D* papers, which include a nice body of work on the RNA polymerase II core promoter as well as advances in diverse areas of chromatin dynamics and transcription. I was also very fortunate to have served on the *G&D* Editorial Board for 13 years (1994–2007). In that capacity, I even had the questionable distinction of being the person who reviewed the most papers for *G&D* in one particular year.

Key components of your success include your keen intellect, judgment, humility, integrity, vision, sense of humor, and appreciation of aesthetics. I have always felt good about submitting papers to *G&D* because I have confidence in you as a scientist, as an Editor, and as a person.

In the spirit of a tribute to you and your work, I will use (or, perhaps more accurately, exploit) this unique opportunity to write two unconventional cover letters. For your perusal, they are on the following pages. Thank you again for a job well done!

Dr. Terri Grodzicker, Editor

*Genes & Development*

Re: Maxims for Scientific Revolutionists

Dear Terri,

I hereby submit a list (*see following page*), which is entitled “Maxims for Scientific Revolutionists,” for consideration for publication in *Genes & Development* in the opinions and rants category.

This list was inspired by the “Maxims for Revolutionists” in “The Revolutionist's Handbook and Pocket Companion,” which is an appendix of the play *Man and Superman* (1903) by G. Bernard Shaw. Perhaps you have read or seen this play. Although much could be said about the play (especially the third act, “Don Juan in Hell”), I will simply say that the “Maxims for Revolutionists” are part of a pamphlet on social revolution that was “written” by the main character, John Tanner, M.I.R.C. (member of the idle rich class).

Because scientists can also be revolutionists (e.g., as described in *The Structure of Scientific Revolutions* by Thomas S. Kuhn), I started to write various Maxims for Scientific Revolutionists. The latest version is attached below. These maxims are a collection of thoughts, not meant to be original, that may be relevant to scientific research. Some items are obvious, and some items are partially explained. Other items might not be obvious but are nevertheless best not explained and left to the imagination.

I am sure that you have written or could easily write lists of maxims on various subject areas. If so, I would be very interested in reading them.

Best wishes,

Jim

Maxims for Scientific Revolutionists

*James T. Kadonaga*

1. Know what you know, and know what you don't know.
2. Have no expectations—you do not know nature.
3. Do not fear the unknown or the contrary.
4. The only stupid thing about a question is not to ask it.
5. A journey into a thick, dark forest will often lead to the greatest enlightenment.
  [Tribute to Dante Alighieri]
6. “I'm following tradition.” = “I'm not thinking about what I'm doing.”
7. Ask simple questions, and then use common sense to answer them.
8. The statement “It can't be done” usually means that it can be done.
9. Think for yourself.
10. If you blend a good wine with a bad wine, you get a lot of bad wine.
11. Timing is really everything.
12. Look forward to what you can gain, rather than fear what you can lose.
13. Practice doesn't necessarily make perfect.
14. Sometimes what you don't do is more important than what you do.
15. Use it or lose it.
16. “The things that are the least cool are always the coolest.” [San Diego surfer Joel Tudor]
17. Call it like you see it.
18. Repeat, repeat, and repeat experiments.
19. “I don't know” can be a good answer.
20. Do not let any interpretation rest on a single data point—apply the Knowles thumb test. [From my Ph.D. thesis advisor, Jeremy R. Knowles, who often said that if the interpretation of an experiment, such as that based on a plot of data points, could be altered if you placed your thumb over one of the data points, you need more data points.]

Dr. Terri Grodzicker, Editor

*Genes & Development*

Re: Landscape of Research

Dear Terri,

I hereby submit a work of digital art, which is entitled “Landscape of Research,” for consideration for publication in *G&D* in the science art category.

From the purely artistic standpoint, I am sure that you would have preferred something like a Chihuly glass sculpture, but this diagram does attempt to make a scientific point. The magenta landscape represents all that could be known, and the red ovals are clusters of specific topics that are currently being investigated. The remaining open areas are subjects that are mostly or completely ignored, but are at least as important as the topics being studied. Hence, this diagram suggests that we have much to learn and that we are placing much of our effort in a few specific areas while ignoring other equally or more important phenomena.

**Figure:**
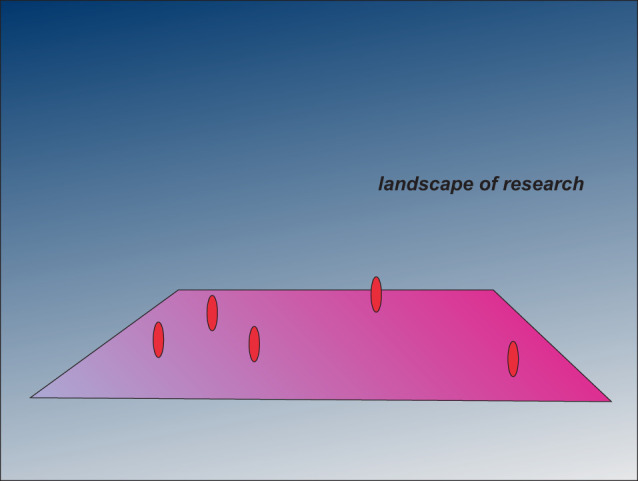

We are now in the year 2023, well into the 21st century! In this regard, we may feel that we know much of what is to be known. The purpose of the Landscape of Research is to suggest the opposite; that is, perhaps we know very little, and don't yet know what we don't know.

This point is, of course, an opinion that may or may not be true. In support of this notion, I suggest that we think of the year 2073, 50 years from now. Would the scientists of 2073 say, “Gosh, back in 2023, they pretty much knew everything!”—or would the scientists of 2073 feel that we had much to learn? Can we even imagine what advances will be made in the next 50 years? The Landscape of Research would likely be as applicable in 2073 as it is today.

At the least, the Landscape of Research might remind us to think about what we know and what we don't know.

Thank you very much for your consideration of this diagram for publication in *G&D.*

With best wishes,

Jim

